# Prevalence and predictive factors of preoperative hypomagnesaemia among adult surgical patients in a large tertiary hospital in Ghana

**DOI:** 10.1186/s12871-015-0116-7

**Published:** 2015-10-06

**Authors:** R. Djagbletey, F. Boni, B. Phillips, Y. Adu-Gyamfi, E. Aniteye, C. Owoo, E. Owusu-Darkwa, A. E. Yawson

**Affiliations:** Department of Anaesthesia, University of Ghana School of Medicine and Dentistry, College of Health Sciences, P. O. Box 4236, Accra, Ghana; Department of Community Health, School of Public Health, College of Health Sciences, University of Ghana, Accra, Ghana

**Keywords:** Preoperative, Serum magnesium, Serum potassium, Serum albumin, Adult surgical, Patients, Laparotomy, Predictive factors

## Abstract

**Background:**

Magnesium is the second most abundant intracellular cation and a co-factor in several reactions involved in the formation and usage of adenosine triphosphate and nucleic acid synthesis. Magnesium deficiency may be as high as 65 % in patients admitted to a medical Intensive Care Unit (ICU). Significant and potentially fatal conditions have been attributed to hypomagnesaemia and it has also been associated with poor prognosis and increased mortality in the critically ill. The study aimed to determine the prevalence and identify the predictive factors of preoperative hypomagnesaemia in adult surgical patients who require an emergency laparotomy.

**Methods:**

This was a hospital based prospective study conducted at the Korle-Bu teaching hospital. General surgical patients between the ages of eighteen and seventy years with a preoperative diagnosis which required emergency laparotomy for management were consecutively enrolled into the study. A total of 102 patients were enrolled in the study. Preoperative total serum magnesium and serum potassium were determined.

Data was summarised utilising simple descriptive statistics (i.e., proportions, ratios and percentages). The Chi-square test was used to determine significant differences or associations between categorical variables, Pearson’s correlation coefficient was used to determine the relationship between continuous variables and predictive factors were determined by multiple regression. Analysis was done in SPSS version 16.

**Results:**

The mean serum total magnesium and potassium were 0.66 ± 0.20 mmol/L and 3.79 ± 0.65 mmol/L respectively. The prevalence of preoperative hypomagnesaemia was found to be 68.0 %. Multiple logistic regression found only hypokalaemia to be a predictive factor (*p*-value of 0.001, odd’s ratio of 9.21 and a confidence interval of 2.42–35.09).

**Conclusion:**

The prevalence of preoperative hypomagnesaemia was high (68.0 %) with hypokalaemia the only predictive factor. Hypokalaemic patients requiring emergency laparotomy are nine times more likely to develop hypomagnesaemia as compared to patients who were not hypokalaemic.

## Background

Magnesium is the second most abundant intracellular cation and a co-factor in more than 300 enzyme regulated reactions, most importantly those involved in the formation and usage of adenosine triphosphate and nucleic acid synthesis [1].

Magnesium deficiency has been demonstrated in 7–11 % of hospitalized patients on general wards [2] but has been found to be as high as 65 % in patients admitted to a medical Intensive Care Unit (ICU) [3]. There is paucity of literature on preoperative hypomagnesaemia. Up to 40 % of hypomagnesaemic patients have been found to have other electrolyte abnormalities [2]. Hypomagnesaemia has been associated with poor prognosis and increased mortality in the acutely ill [4, 5].

Known causes of hypomagnesaemia include: Reduced dietary intake, poor gastrointestinal absorption, increased losses from the gastrointestinal tract (as in diarrhoea, vomiting and laxative use), increased renal losses (as in congenital/acquired renal tubular defects, diabetes mellitus and alcoholism) and drugs (diuretics, angiotensin converting enzyme (ACE) inhibitors, aminoglycosides, amphotericin, cyclosporine, cisplatin) [1, 6]. Previous studies have indicated magnesium intake to be lower and the prevalence of hypomagnesaemia higher among populations with low socio-economic background [7].

Deficiency of magnesium causes weakness, muscle cramps, cardiac dysrhythmia and increased irritability of the nervous system with tremors, athetosis, jerking, nystagmus and an extensor plantar reflex. In addition, it may cause confusion, disorientation, hallucination, depression, hypertension, tachycardia and tetany [1, 8].

Hypomagnesaemia is associated with changes in the electrocardiogram (ECG) of patients; widening of the QRS complex and peaking of T waves when moderate, whereas more severe magnesium depletion can lead to prolongation of the PR and QT intervals, progressive widening of the QRS complex, and diminution of the T wave. It is often associated with supraventricular and ventricular tachy-arrhythmias. Of these, Torsade de pointes a polymorphic ventricular tachycardia with prolongation of QT interval is often known to be associated with magnesium deficiency [1, 9].

It is unclear if cardiac arrhythmias associated with hypomagnesaemia are due solely to serum magnesium levels or whether the often concomitant hypokalaemia and other electrolyte disturbances also play a role [1].

The importance and the role of magnesium in medicine have advanced considerably in recent years. It is now generally accepted that magnesium is a critical nutrient and that magnesium deficiency has adverse effects on a variety of physiological processes. It has therefore become evident that magnesium deficiency should be avoided in the perioperative period and in critical care [10].

This was a prospective cohort study to profile risk factors associated with preoperative hypomagnesemia in adult surgical patients who require an emergency laparotomy.

## Methods

This was a hospital based prospective study conducted at the Korle-Bu teaching hospital (KBTH) between November 2012 and April 2013. The Korle-Bu Teaching Hospital, a tertiary health care facility in Ghana, was the survey site. The KBTH has a bed capacity of 2000 (total number of beds available for admitted patients in all departments/units of the hospital) and over 3000 staff. In 2010, 29,757 clients were seen on the average per month, average daily outpatient attendance was 1500 and average daily admission was 150.

All general surgical patients between the ages of 18–70 years requiring emergency abdominal surgery admitted at Korle-Bu Teaching Hospital who gave informed consent were selected consecutively and enrolled in the study. The sample size for this prospective survey was determined based on the hypothesis that ‘perioperative surgical patients in the Korle-Bu Teaching Hospital do not experience hypomagnesaemia’, and a demonstrated magnesium deficiency in hospitalized patients on general wards as (7–11 %) [2], at the 95 % confidence level and power of 90 %, a total of 102 patients were enrolled. Pregnant women and patients with chronic kidney disease were excluded from the study.

A self-designed questionnaire was used to collect socio-demographic and medical data of the patient including the intra-operative diagnosis. A six (6) millilitre blood sample was taken, into gel separator tubes without the use of a tourniquet, just before induction of anaesthesia for the determination of the preoperative serum concentrations of magnesium, potassium and albumin.

Agitation or shaking of the whole blood was avoided to prevent haemolysis of the sample. Samples were centrifuged at 5000 revolutions per minute to obtain the serum. All laboratory investigations were done at the National Cardio-Thoracic Centre (NCTC) laboratory, Korle-Bu.

Serum total magnesium was determined by photometry using Xylidyl blue. Serum total magnesium and albumin was determined using Eos Bravo forte auto analyser, from Hospitex Diagnostics, Italy. Serum potassium concentration was determined by direct ion selective method using an auto analyser (Starlyte^TM^ V ISE, Alfa Wassermann, from Diagnostic Technologies LLC, Netherlands).

The reference ranges of the National Cardio Thoracic Centre laboratory [11] for serum potassium, total magnesium were 3.5–5.0 mmol/L and 0.74–1.03 mmol/L respectively. Results were categorised as “normo”, if within the reference range, “hypo” if less than the lower limit of normal and “hyper” if higher than the upper limit of normal.

Two samples were excluded from laboratory analysis because one was haemolysed and the other was of inadequate volume. Patients found to have hypomagnesaemia were appropriately treated during the perioperative period.

### Data analysis

Data was captured using Microsoft Access 2007 Database. Analysis was done with Statistical Package for the Social Sciences (SPSS) software version 16. Survey data were analysed by simple descriptive statistics (i.e., proportions, ratios and percentages) and summarized in tables. The Chi-square test was used to determine significant differences or associations between categorical variables, Pearson’s correlation coefficient was used to determine the relationship between continuous variables and predictive factors were determined by multiple regression at the at the 95 % confidence interval. A *p*-value of < 0.05 was judged as significant.

### Ethical issues

Ethical Approval for the survey was obtained from the Ethical and Protocol Review Committee of University of Ghana Medical School (Protocol Identification Number: MS-Et/M.2 - P 5.3/2011-12) Clearance was also received from the Management of the Korle-Bu Teaching Hospital and Heads of Clinical units where survey was conducted.

## Results

### Socio-demographic data

A total of 102 patients were enrolled in the study with a mean age of 42.75 ± 15.67 years. The female: male ratio was thus 1: 1.55 the age-sex distribution is as shown in Table 1. Other demographic data recorded were educational level and occupation.

**Table 1:** Age - Sex distribution of patients undergoing emergency laparotomy, Korle-Bu Teaching Hospital, Ghana

Age (years)	Sex		Total
	Male	Female	
	Frequency (Percentage)	Frequency (Percentage)	Frequency (Percentage)
<20	3(2.9)	3(2.9)	6(5.9)
21–30	16(15.7)	9(8.8)	25(24.5)
31–40	11(10.8)	9(8.8)	20(19.6)
41–50	10(9.8)	8(7.8)	18(17.6)
51–60	8(7.8)	5(4.9)	13(12.7)
61–70	14(13.7)	6(5.9)	20(19.6)
Total	62(60.8)	40(39.2)	102(100.0)

Nearly half the patients (45.1 %) had received basic education, 27.5 % had secondary, and 19.6 % had received tertiary education. Only eight patients representing 7.8 % of patients had no formal education. The majority of the patients were either traders (29.4 %) or artisans (16.7 %). Professionals (pharmacists, administrators, bankers and secretaries) form 13.7 % of the patients. Others in other occupations such as pastors, footballers and farmers formed 7.8 % of patients.

### Health status assessment

The mean duration of illness was 5.13 ± 4.97 days with a range of 5 h to 23 days. Fifty-seven patients (55.9 %) had history of vomiting whilst 5.9 % had history of diarrhoea. Thirty-nine patients (38.2 %) admitted to the use of alcohol.

Hypertension was the commonest (16.7 %) known co-morbid condition among enrolled patients. Four (3.9 %) patients were both hypertensive and diabetic. Two patients were known asthmatics. None of the patients enrolled were known sickle cell disease patients.

Nine patients, representing 8.8 % were on either loop or thiazide diuretics and two patients were on angiotensin converting enzyme (ACE) inhibitors. No patient was on an aminoglycoside, amphotericin, cyclosporine or cisplatin.

Majority of the patients were ASA (American Society of Anesthesiologists) physical status II_E_ (37.2 %). A third of the patients were ASA III_E_, whilst 22.5 % were ASA I_E_. Only 4.4 % were ASA IV_E_. No patient with ASA V_E_ was enrolled in the study.

The most frequent diagnosis encountered was either an obstructed or a strangulated hernia (27.5 %) followed closely by acute or complicated appendicitis (21.6 %) and intestinal obstruction (19.6 %). Other conditions diagnosed included pancreatitis, anastomotic leakage, bleeding duodenal ulcer and gall bladder empyema as shown in Table 2.

**Table 2:** Health Status Assessment of patients undergoing emergency laparotomy, Korle-Bu Teaching Hospital, Ghana

	Frequency	Percentage (%)	Mean duration
History			
Diarrhoea	6	5.9	2.50 ± 1.05 days
Vomiting	57	55.9	2.42 ± 2.91 days
Alcohol use	39	38.2	11.49 ± 10.09 years
Co-morbid condition			
Hypertension	17	16.7	
Diabetes mellitus	4	3.9	
Hypertension + diabetes	4	3.9	
Asthma	2	2.0	
Medications			
Diuretics (Loop and Thiazide)	9	8.8	
ACE inhibitors	2	2.0	
Intra-operative diagnosis			
Acute & complicated appendix	22	21.6	
Intestinal obstruction	20	19.6	
Obstructed / strangulated hernia	28	27.5	
GIT perforation	13	12.7	
Intra-abdominal abscess	8	7.8	
Others	11	10.8

**Table 3:** Descriptive and reference range for serum magnesium and potassium

	*N*	Reference range	Minimum	Maximum	Mean	Std. deviation
Potassium(mmol/L)	101	3.5–5.0	2.2	5.5	3.79	0.65
Magnesium(mmol/L)	100	0.74–1.03	0.11	1.08	0.66	0.20

### Laboratory results

The mean serum total magnesium was 0.66 ± 0.20 mmol/ whilst the mean serum potassium was 3.79 ± 0.65 mml/L as shown in Table 3. Thirty per cent of patients had normal magnesium levels. The prevalence of hypomagnesaemia among the patients was 68.0 %, while 2.0 % were hypermagnesaemic (Table 4).

**Table 4:** The distribution of serum magnesium status of patients

	Frequency	Percentage (%)
Magnesium status		
Hypomagnesaemia	68	68.0
Normo-magnesaemia	30	30.0
Hypermagnesaemia	2	2.0
Total	100	100.0

**Fig. 1:**
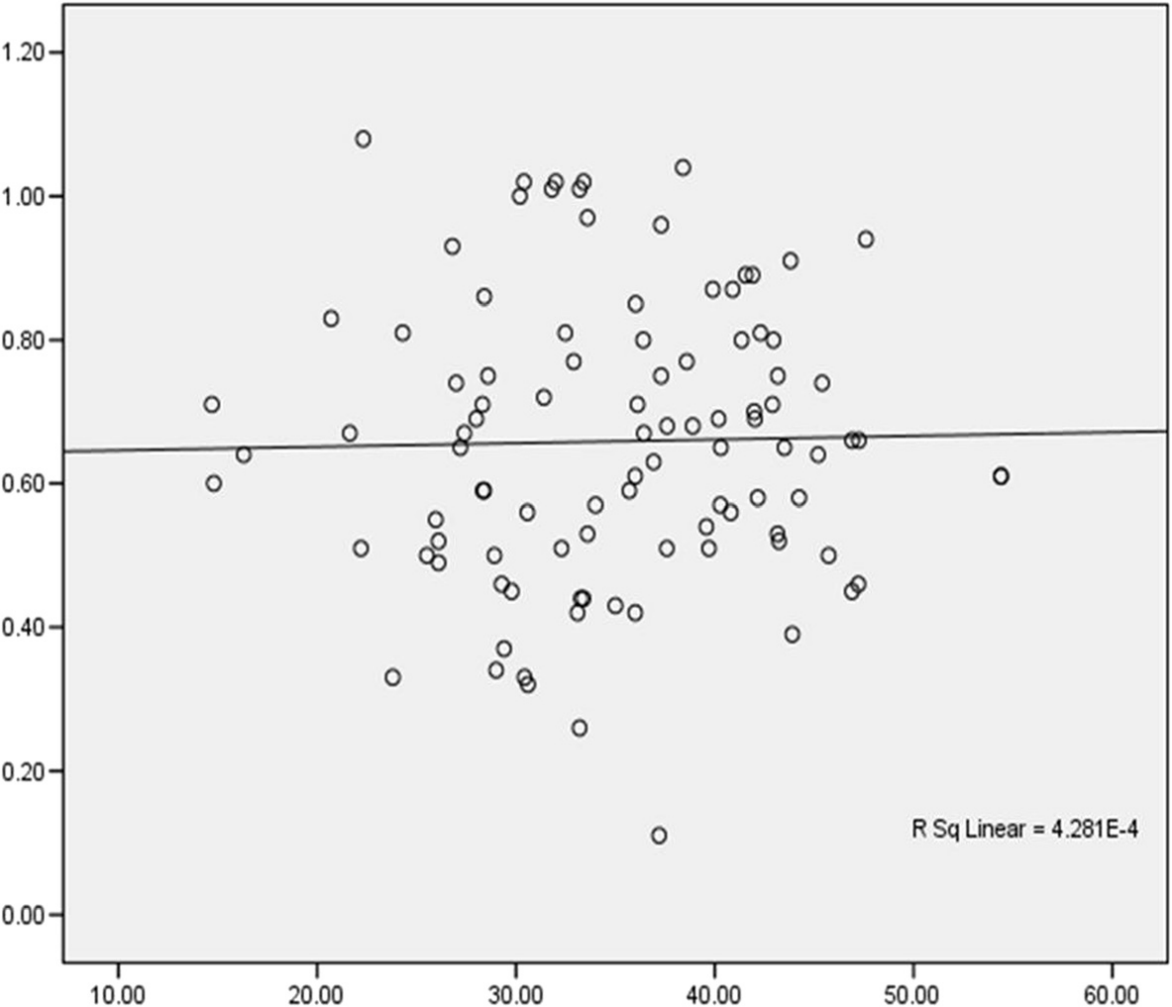
Correlation between serum albumin and serum total magnesium levels. X-axis: Serum albumin (g/dl). Y-axis: Serum total magnesium (mmol/L)

There was no correlation between serum total magnesium and serum albumin (Pearson’s correlation coefficient = 0.02, R2 = 0.000, *p* = 0.839) as shown in Fig. 1.

There was significant association between the potassium status and magnesium status as depicted by Chi-square of 10.99, *p*-value = 0.027 as shown in Table 5.

**Table 5:** Magnesium status by potassium status

		Magnesium status			Total	*χ* ^2^ value and (df)	*p*-value
		Normo	Hypo	Hyper			
Potassium status	Normo	24 (24.0)	35 (35.0)	2 (2.0)	61 (61.0)	10.99 (4)	0.027
	Hypo	4 (4.0)	31 (31.0)	0 (0.0)	35 (35.0)		
	Hyper	2 (2.0)	2 (2.0)	0 (0.0)	4 (4.0)		
Total		30 (30.0)	68 (68.0)	2 (2.0)	100 (100.0)		

Of the 35 patients that were hypokalaemic, 31 (88.6 %) were hypomagnesaemic. Of the 68 patients that had hypomagnesaemia, 31 (45.5 %) were hypokalaemic.

### Predictive factors

Only hypokalaemia was found to predict hypomagnesaemia (Wald’s score =10.60, *p*-value =0.001, odd’s ratio = 9.21, confidence interval = 2.42–35.09) as shown in Table 6.

**Table 6:** Multiple logistic regression between hypo/normo magnesium values against Predictive factors

Factors	*p*-value	Odd’s ratio	Confidence interval	
			Lower	Upper
Sex	0.715	0.82	0.28	2.39
Duration of illness	0.658	1.00	0.99	1.00
Time of last meal	0.499	1.85	0.31	10.93
Diarrhoea	0.888	0.86	0.10	7.39
Vomiting	0.592	0.76	0.27	2.10
Alcohol	0.449	0.66	0.23	1.92
Hypertension	0.503	0.42	0.03	5.42
Diabetes Mellitus	0.486	0.34	0.02	7.01
Asthma	0.954	1.09	0.06	20.66
Diuretics	0.333	3.99	0.24	65.47
ASA	0.886	0.95	0.49	1.86
Surgical diagnosis	0.373	0.84	0.56	1.24
Hypokalaemia	0.001	9.21	2.42	35.09

## Discussion

### Prevalence of hypomagnesaemia

The prevalence of preoperative total serum hypomagnesaemia, among adult surgical patients requiring an emergency laparotomy, from the study was found to be 68.0 %. This was far higher than found in similar studies among hospitalised patients in general (7–11 %) but consistent with that recorded among patients admitted to post-operative (61 %) and medical (65 %) ICUs [2, 3, 12].

Most patients requiring surgical care in the West African sub-region usually present late, having delayed in seeking medical attention [13]. This results in significant number of patients presenting in severely ill states as was found from the study. Forty point two percent (40.2 %) of patients presented with ASA status of III_E_ and IV_E_. Such severely ill patients may be in significant stress from the underlying surgical pathologies and the abdominal pain which is a common presenting feature of patients requiring emergency laparotomy.

When patients present at medical facilities, there are delays at the peripheral hospitals prior to referral and also significant delays within the referral hospitals [14]. The anxiety caused by such delays adds to the stress of these patients.

The stress from the surgical pathology, pain and anxiety may increase circulating catecholamine levels in patients requiring an emergency laparotomy. High concentration of circulating catecholamine is known to cause an intracellular shift of magnesium resulting in serum hypomagnesaemia [1]. This may be one of the factors contributing to the high prevalence of hypomagnesaemia observed.

From population level studies, magnesium intake has been found to be lower in people with low socio-economic background [12]. From this study, 52.9 % of patients were without secondary education and 46.1 % were either traders or artisans. Such patients may therefore be more prone to developing hypomagnesaemia as they may have a low magnesium intake.

Using the highest level of education as a proxy of socio-economic status, this study did not find magnesium level to be significantly associated with the socio-economic status of the patients we studied, contrary to findings from population studies [7].

The principal constituents of most Ghanaian dishes are: corn flour, cassava and polished rice which are extremely poor in magnesium content [15]. The corn flour used in Ghana is mainly from whole grain corn and may thus contain reasonable amounts of magnesium. The meals prepared from such flour usually involve cooking and prolonged boiling, leading to significant magnesium loss [1]. Although beans, legumes and green leafy vegetables which have high magnesium content form part of our diet, these are usually boiled or cooked depleting their magnesium content significantly. Poor dietary intake of magnesium could be a contributing factor to the high prevalence of hypomagnesaemia observed.

Similar to other studies [1, 16] done in the critically ill, no association was found between magnesium status and sex.

### Predictive factors

#### Serum potassium

In this study, majority (88.6 %) of the hypokalaemic patients were also hypomagnesaemic. A multiple logistic regression found only preoperative serum potassium level to be of significant predictive value for hypomagnesaemia in adult general surgical patients requiring emergency laparotomy (*p*-value = 0.001). With an odd’s ratio of 9.21, it implies that hypokalaemic adult general surgical patients requiring emergency laparotomy are nine times more likely to develop hypomagnesaemia as compared to patients who were not hypokalaemic.

Only 45.5 % of hypomagnesaemic patients were also hypokalaemic. Thus determining magnesium levels only among hypokalaemic patients would risk missing majority of the hypomagnesaemic patients. It would thus be prudent to routinely test for magnesium in all adult patients who require emergency laparotomy and not just in those who are hypokalaemic.

#### Diarrhoea and vomiting

In the study, diarrhoea (5.9 %) was not as common a presentation among surgical patients requiring laparotomy as vomiting (55.9 %). This may be attributed to the fact that a lot of the cases (47.1 %) had some form of bowel obstruction. Upper gastro-intestinal tract obstruction is more commonly associated with vomiting. Neither history of diarrhoea nor vomiting could predict hypomagnesaemia. Perhaps a mean duration of less than three days might not have been long enough for diarrhoea and vomiting to have caused significant changes in serum magnesium levels.

Naso-gastric drainage constitutes part of fluids and electrolytes (including magnesium) lost from the body. Most patients in this study (94.1 %) had naso-gastric tubes inserted at time of admission. Safavi and Monarmand in a similar study of critically ill patients admitted to an intensive care unit found that naso-gastric drainage was an independent risk factor for the development of hypomagnesaemia [5]. This may have also contributed to the prevalence of hypomagnesaemia observed.

#### Surgical diagnosis

Significant association has been reported between hypomagnesaemia and oesophageal surgeries [17]. Szpetnar and co-workers observed preoperative total serum hypomagnesaemia in patients with colorectal and small bowel cancer [18]. Other studies have shown that total serum magnesium decreases after gastro-intestinal surgeries [5]. Similar to finding by Ohene-Yeboah in 2004 at the Komfo Anokye Teaching Hospital, Kumasi, Ghana [19], the three most common diagnosis for emergency laparotomy in this study were: acute and complicated appendicitis, intestinal obstructions and strangulated/obstructed hernia. Though the three top diagnoses contributed 72.0 % of hypomagnesaemic patients, surgical diagnosis did not predict hypomagnesaemia. This may be due to the fact that only few patients in this study had a preoperative diagnosis of bowel malignancy which is known to impair diet and is associated with hypomagnesaemia.

#### Time of last meal

Surgery and dietary regimen immediately (48 h) before surgery have been found to be important causes of hypomagnesaemia [16]. This study thus investigated the effect of the time of last meal on the serum magnesium status of patients. The time of last meal was not found to predict hypomagnesaemia (*p*-value >0.05). The mean duration of the time of last meal was 22.67 ± 21.83 h. This period of time without magnesium intake might not have been long enough to have caused abnormalities in serum total magnesium levels.

#### Alcohol

Alcohol use is a known cause of hypomagnesaemia. Other mechanisms contributing to magnesium depletion include: poor nutritional status, magnesium loss through vomiting and diarrhoea, malabsorption and renal tubular dysfunction resulting in increased magnesium loss [1]. Similar to findings of a study done in critically ill medical patients, this study did not find a history of alcohol use to significantly influence the magnesium status of patients [20]. This observation may be due to the fact that alcohol consumption among the patients was low with a mean of 5.29 ± 9.33units of alcohol consumed per week.

#### Medical conditions

Medical conditions such as hypertension, diabetes and asthma have been associated with hypomagnesaemia. A study done in patients admitted to an intensive care unit found hypertension and diabetes to be predictive for hypomagnesaemia [21]. In this study, however, hypertension, diabetes and asthma were not found to be predictive factors for hypomagnesaemia.

#### Medications

History of diuretic use has been found to be a predictive factor for hypomagnesaemia in studies done on patients admitted to intensive care units [12, 16]. In this study however, a history of diuretic use was not found to be a predictive factor for hypomagnesaemia.

#### Albumin

About a third of serum magnesium is bound to proteins especially albumin, changes in serum albumin level, therefore, may affect serum total magnesium concentration [1]. This study found no correlation between serum total magnesium and serum albumin as has been reported in other similar studies [12, 17]. A correction of the total magnesium concentrations for serum albumin was however not done in the current analysis to investigate the relationship further.

Thus neither, sex, duration of illness, time of last meal, history of diarrhoea, vomiting, alcohol use, hypertension, diabetes, used of diuretics and surgical diagnosis were not found to significantly predict preoperative serum total hypomagnesaemia in adult general surgical patients requiring emergency laparotomy. The relatively small sample used could have reduced the power of the study and hence the inability to detect predictive factors for hypomagnesaemia.

In this study, three patients had cardiac dysrhythmia which resolved with correction of serum magnesium levels. One patient had paroxysmal ventricular tachycardia, another had ventricular ectopic beats and the third had supra-ventricular tachycardia.

Most hypomagnesaemic patients (97.1 %) were therefore asymptomatic similar to findings of Chernow et al. [12]. This may be due to the fact that serum magnesium levels may not reflect total body or tissue magnesium levels [22, 23]. Furthermore, most symptoms of hypomagnesaemia are non-specific [12] and may often be attributed to the other electrolyte imbalances (hypokalaemia, hypocalcaemia and hyponatraemia among others) which are frequently associated with hypomagnesaemia [2].

Patients presenting for emergency laparotomy had fluid resuscitation with either normal saline or Ringer’s lactate prior to surgery. Varying amounts of these magnesium-free fluids were used during resuscitation depending on the degree of dehydration of the patient. If significant volumes of such magnesium-free fluids are infused, it could lead to dilution hypomagnesaemia [1] and this may contribute to the high prevalence of hypomagnesaemia observed. The type and volume of fluids used in resuscitation were not determined in this study. Ringer’s Lactate contains potassium (4 mmol/L) and this could have affected the serum potassium levels determined.

The current analysis did not determine patients who were transfused preoperatively and the amount of blood and blood products transfused. Citrated blood and blood products would cause chelation of magnesium and may have contributed to the hypomagnesaemia observed; results from the analysis should therefore be interpreted in this context.

It appears that magnesium deficiency is not often thought about and therefore symptoms of hypomagnesaemia may often not be noticed or may be attributed to other causes. This may lend credence to the view that magnesium is the “forgotten cation” in clinical practice [24]. Due to the high prevalence of hypomagnesaemia observed and that most hypomagnesaemic patients were asymptomatic, routine determination of total serum magnesium levels and correction of abnormalities detected may be required in adult general surgical patients who require emergency laparotomy. More so for the patients found to be hypokalaemic as they have a higher risk of developing hypomagnesaemia.

The results of this study, however, should thus be interpreted with caution in view of the limitations enumerated below.

Limitations of the study were that no control group were included to compare the patients to in terms of total serum magnesium levels and that laboratory reference ranges used were not based on population studies done in Ghana. Ghanaians may have different reference ranges for the investigations conducted however, national level references ranges are not available. Clinical management of patients in the Hospital are based on these reference ranges and this baseline survey will be important for future larger surveys. In addition, serum calcium and phosphate levels, which are known to affect serum total magnesium levels, were not determined. The amount and type of intravenous fluids used, blood and blood products transfused were also not determined. Conclusions from this study should thus be interpreted within the confines of this limitation.

## Conclusions

The prevalence of serum total hypomagnesaemia among adult general surgical patients requiring emergency abdominal surgery was high (68.0 %). Sex, duration of illness, time of last meal, history of diarrhoea, vomiting, alcohol use, hypertension, diabetes, use of diuretics and surgical diagnosis were not found to significantly predict preoperative serum total hypomagnesaemia in adult general surgical patients requiring emergency laparotomy.

Only preoperative serum potassium levels were significantly predictive of hypomagnesaemia in adult general surgical patients requiring emergency laparotomy. Hypokalaemic adult general surgical patients requiring emergency laparotomy were found to be nine times more likely to develop hypomagnesaemia as compared to patients who were not hypokalaemic.

### Recommendations

1. Preoperative serum magnesium levels should be routinely determined and abnormalities corrected in all general surgical patients requiring an emergency laparotomy.
2. Adult general surgical patients requiring emergency abdominal surgery who are hypokalaemic are likely to be hypomagnesaemic, Therefore magnesium replacement therapy should be considered together with the potassium replacement when there is adequate urine output.
3. Studies to determine the normal magnesium levels and the incidence of hypomagnesaemia in the general Ghanaian population are required.
4. Further studies with larger sample sizes to investigate predictive factors for hypomagnesaemia in preoperative surgical patients are needed.

## Abbreviations

ICU: Intensive Care Unit
KBTH: Korle-Bu Teaching Hospital
NTCC: National Cardio-thoracic Centre
mmol/L: millimoles per litre
mmol/Kg: millimoles per kilogram body weight
ACE: Angiotensin Converting Enzyme
ASA: American Association of Anesthesiologists
